# 

**Published:** 1898-06-25

**Authors:** 


**Notices of Births, Deaths, and Marriages** are inserted in this column at a charge of 2s. 6d. for an announcement not exceeding four lines.

### NOTICE TO CORRESPONDENTS.

All MS., Letters, Books for Review, and other matters intended for the Editor should be addressed The Editor, "The Hospital," The Hospital Building, 28 & 29, Southampton Street, Strand, W.C.

The Editor cannot undertake to return rejected MS., even when accompanied by stamped directed envelope.

**Notice to Advertisers and Subscribers.**

All Advertisements, Orders for Copies of The Hospital, and Business Communications should be addressed to **THE MANAGER, (Not to The Editor),** The Hospital, The Hospital Building, 28 & 29, Southampton Street, Strand, London, W.C.

The Cost of Subscription, post free, is as follows:—Per week, 2½d. per quarter, 2s. 8d.; per half-year, 5s. 3d.; per annum, 10s. 6d. Foreign:—Per annum, 15s. 2d.; payable, in all cases, in advance.

**NOTICE.—Vol. XXIII. of "The Hospital" (October, 1897, to March, 1898), in handsome cloth binding, is now on sale at the Office of this Journal, price 7s. 6d. Cases for binding the Numbers contained in this Volume, 1s. 6d. Index to Vol. XXIII., 2½d., post free.**

**The Monthly Part for July will be ready on July 1. Price 6d., by post 8d.**

### BOOKS RECEIVED.

Charles Griffin and Co.

"Year Book of Scientific and Learned Societies."

J. R. Williams.

"Report on the Health of Liverpool." By E. W. Hope, M.D., D.Sc.

Scientific Press.

"Burdett's Hospital and Charities, 1898. The Year Book of Philanthropy, and Hospital Annual."

### The Student of Medicine.

### APPOINTMENTS.

J. Blumfeld, M.D., B.C.Cantab., appointed Anæsthetist to the Grosvenor Hospital for Women and Children. W. J. Bowden, M.B., Ch.B.Vict., appointed Medical Officer for the Glossop Union Infirmary. Ada M. Browne, L.S.A.Lond., appointed Senior Assistant Anæsthetist to the New Hospital for Women, Euston Road, London. J. Cantlie, M.A., M.B., F.R.C.S., appointed Surgeon to the West End Hospital for Nervous Diseases, Welbeck Street. S. R. Christopher, M.B., Ch.B.Vict., appointed Assistant Medical Officer of the Mill Road Infirmary of the West Derby Union. W. T. Davies, M.R.C.S.Eng., L.R.C.P., appointed Medical Officer for the Second and Third Districts of the Hertford Union. R. Dick, M.B., Ch.M.Syd., D.P.H., appointed Medical Officer of Health for the Combined Hunter River Districts, Newcastle, N.S.W. J. G. Forbes, M.B., C.M.-Edin., appointed Assistant Resident House Surgeon to the Cardiff Infirmary. E. B. Fuller, M.B., C.M.Edin., F.R.C.S.E., appointed to the Honorary Visiting Staff of the Somerset Hospital, Cape Town, Cape Colony. H. P. Godfrey, M.B.Melb., F.R.C.S.-Eng., appointed Health Officer at Bardock, Western Australia. B. B. Gough, M.R.C.S., L.R.C.P.Lond., appointed House Surgeon to the Burton-on-Trent Infirmary. T. P. Greenwood, M.B., C.M., B.Sc.Edin., appointed Third Assistant Medical Officer to the County Asylum, Fareham, Hants.

